# Autoimmune thyroid disease and urticarial vasculitis: is there a significant association?

**DOI:** 10.1186/s13223-019-0339-0

**Published:** 2019-04-18

**Authors:** Ivan Cherrez-Ojeda, Emanuel Vanegas, Valeria L. Mata, Miguel Felix, German D. Ramon, Sofia Cherrez, Annia Cherrez

**Affiliations:** 1 Universidad Espíritu Santo, Km. 2.5 vía La Puntilla, Samborondón, Zip code: 0901-952 Ecuador; 2 Respiralab, Respiralab Research Group, Guayaquil, Ecuador; 3 Allergy Section, Hospital Italiano Regional del Sur, Bahía Blanca, Argentina; 4 University of Heidelberg, Heidelberg, Germany; 5 Clinic and Policlinic for Dermatology and Venereology, University Medical Center Rostock, Rostock, Germany

**Keywords:** Urticarial vasculitis, Thyroid autoimmunity, Chronic spontaneous urticaria, Autoantibody, Immune response

## Abstract

Little is known about the association of urticarial vasculitis (UV) with thyroid autoimmunity. The latter has been mostly described in the setting of patients with chronic spontaneous urticaria (CSU). In this letter to the editor, we compare UV and CSU through retrospective analyses, which reveal that 41.7% patients with UV presented antithyroperoxidase (anti-TPO) and/or antithyroglobulin antibody (ATA) above the reference range, while only 4% patients with CSU had these antibodies elevated. There is a moderately strong association assessed by the Phi coefficient (φ = 0.420, p = 0.004). Further research is needed to appropriately address the relationship between UV and thyroid autoimmunity and explore any potential underlying pathophysiological process between both diseases.

Urticarial vasculitis (UV) is identified as a clinicopathologic entity that involves clinical features of urticaria and histopathologic findings compatible with a cutaneous leukocytoclastic vasculitis of small vessels with fibrinoid deposits. UV represents a spectrum of diseases that differ in severity, ranging from an urticaria with minimal vasculitis to a systemic disease that can lead to serious organ-specific complications. Certainly, urticarial vasculitis is an underdiagnosed disease, to the extent that its incidence may vary from 3 to 20% [1]. This is due to the lack of a consensus in medical literature upon a disease that manifests diversely, with a definite diagnosis that relies on a high-tech procedure such as biopsy.

Several findings may facilitate its diagnosis, such as abnormal acute phase reactants, hypocomplementemia, positive antinuclear antibodies (ANA) and other laboratory tests that have been extensively described, as well as common associations with other clinical conditions such as systemic lupus erythematosus (SLE), lupus-like disease and Sjogren’s Syndrome [1, 2]. However, little is known of how UV associates with thyroid autoimmunity. The latter has been mostly described in the setting of patients with chronic spontaneous urticaria (CSU), with evidence suggesting that CSU can improve in response to treatment with levothyroxine or other thyroid drugs [3]. In this report we discuss the potential implication of screening UV patients for abnormally elevated IgG anti-thyroid Abs (AAbs), as this feature has been described as a predictor of hypothyroidism in euthyroid patients [4].

We carried out a retrospective analysis from 2005 to 2016 involving 98 patients diagnosed with either chronic spontaneous urticaria or urticarial vasculitis at Respiralab Urticaria Center, Guayaquil-Ecuador. Demographic and clinical variables such as age, sex, years with the disease, type of urticaria, thyroid function tests and antithyroid antibodies were collected using medical records from the institution. The diagnostic definitions used for chronic spontaneous urticaria and urticarial vasculitis are summarized in Table 1 [5–8]. All patients with clinical features suggestive of urticarial vasculitis were confirmed by biopsy. Thyroid function tests (T3, T4, TSH) were categorized into normal, below normal or above normal, while AAbs (antithyroperoxidase, antithyroglobulin antibodies) where dichotomized as absent or present.

**Table 1:** Definition of chronic spontaneous urticaria and urticarial vasculitis

Chronic spontaneous urticaria
Spontaneous development of wheals, angioedema, or both for > 6 weeks. Wheals have the following characteristics
1. Central swelling surrounded by reflex erythema.
2. Itching, sometimes burning sensation
3. Skin returning to normal appearance within 30 min to 24 h
Urticarial vasculitis
Clinical manifestations of urticaria characterized by wheals that are painful, burning, and tender in sensation. Lesions tend to last > 24 h and are associated with faint residual hyperpigmentation. Biopsy is needed to confirm the diagnosis, with the following findings
1. Inflammatory injury to capillaries and postcapillary venules
2. Leukocytoclasis with fibrinoid deposits as signs of direct vessel damage

References: [5–8]

Descriptive analyses (frequency, percentage, standard deviation) were carried out for demographic and clinical variables. The Chi square test was applied to analyze the fit of distribution and to measure association with the Phi coefficient between the diagnosis and the presence/absence of AAbs. Crude analysis with binomial logistic regressions between diagnosis and antithyroid antibodies were performed. All statistical analyses were carried out using SPSS v24.0 (IBM, Armonk, NY, USA). *P* < 0.05 was considered significant.

Descriptive analyses from the 98 patients involved showed that 25.5% (n = 25) were males and 74.5% (n = 73) were females. The mean age of the sample studied was 38.12 years (SD 15.97), and the mean length of the disease since diagnosis was 1.49 years (SD 2.19). Concerning the disease, 87.8% (n = 86) patients were diagnosed with chronic spontaneous urticaria and 12.2% (n = 12) with urticarial vasculitis (Fig. 1).

**Fig. 1:**
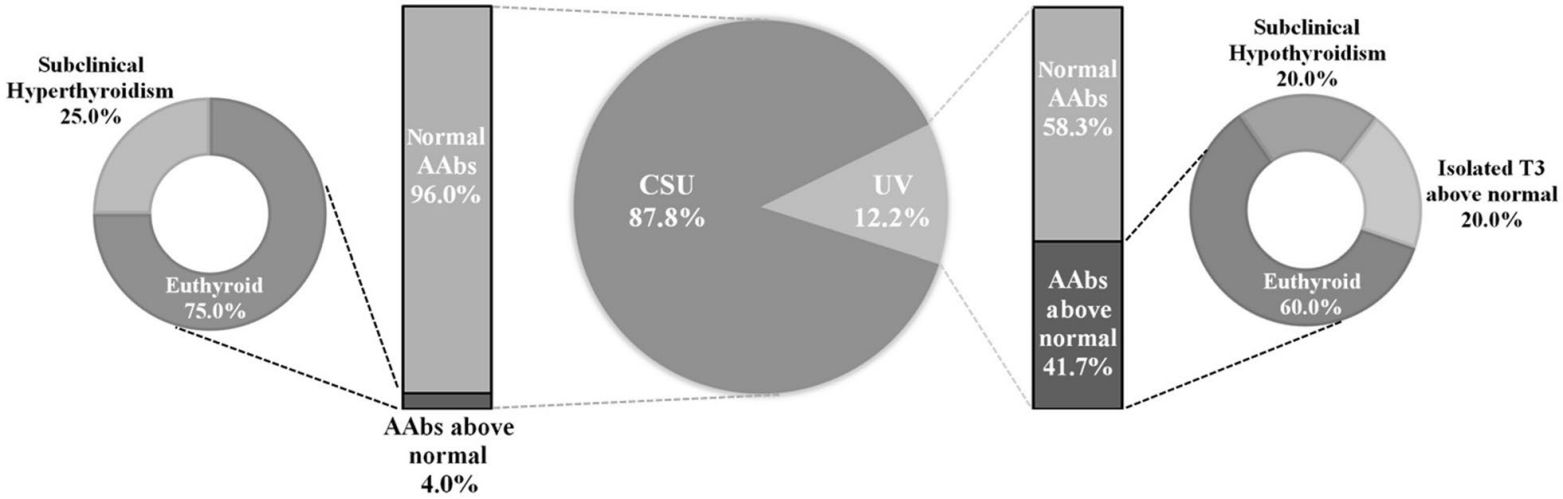
Anti-thyroid antibodies (AAbs) and thyroid function profile on chronic spontaneous urticaria (CSU) and urticarial vasculitis (UV) patients

From 12 patients with urticarial vasculitis, 41.7% (n = 5) presented antithyroperoxidase (anti-TPO) and/or antithyroglobulin antibody (ATA) above the reference range (p = 0.564), while only 4% (n = 4) out of the 86 patients with CSU had these AAbs elevated (p = 0.000). The observed frequency for patients with AAbs above normal with urticarial vasculitis was greater than the expected value, while for CSU patients it was less than expected, with a moderately strong association assessed by Phi coefficient (φ = 0.420, p = 0.004). Patients with UV were 14.64 times more likely to have abnormally elevated levels of AAbs than CSU patients (p = 0.001). Regarding complement 66.6% (n = 8) of patients had low levels (defined by low C3 and C4), and 8.3% (n = 1) had normal levels. Unfortunately, 25% (n = 3) of patients did not have complement measurements. There were no UV patients presenting with associated SLE, or related collagen disorder.

With respect to thyroid profile on individuals with elevated AAbs, we found that 75% (n = 3) patients with CSU were euthyroid, while 25% (n = 1) were compatible with subclinical hyperthyroidism. On the other hand, 60% (n = 3) cases diagnosed with UV were euthyroid, while 20% (n = 1) were compatible with subclinical hypothyroidism and another 20% (n = 1) had T3 levels above normal with T4 and TSH in the reference range.

Annual reassessment of thyroid function and AAbs levels in UV patients may be recommended, since levels of AAbs have shown to predict the future development of hypothyroidism in an euthyroid general population [4]. In our sample, all patients with elevated AAbs were euthyroid, except for three patients, of which two were compatible with subclinical hypothyroidism. Currently, it is known that the presence of AAbs in the patient with CSU does not associate with abnormal thyroid function, since most patients are usually euthyroid [9]. However, in a minority of patients with Hashimoto’s thyroiditis, administration of levothyroxine may improve chronic urticaria symptoms, hypothetically highlighting a common pathogenesis between such entities [10]. It is not known if treating thyroid disease in UV patients might resolve urticaria symptoms in some degree, a point we consider of interest for future research.

As relevant limitations to our study we must mention the small sample size and retrospective nature. Nevertheless, it would be interesting to develop new research to appropriately address the relationship between UV and thyroid autoimmunity, to explore if there is an underlying pathophysiological process between both diseases, and to determine if early treatment of thyroid disease is recommended. Only this data would, in clinical practice, support the screening of AAbs in UV patients.

## Abbreviations

UV: urticarial vasculitis
ANA: antinuclear antibodies
SLE: systemic lupus erythematosus
CSU: chronic spontaneous urticaria
AABs: anti-thyroid antibodies
anti-TPO: antithyroperoxidase
ATA: antithyroglobulin antibody
